# Ecocardiografia Guiando Tratamento Percutâneo de Cardiomiopatia Hipertrófica Obstrutiva: Navegar (em Águas Conhecidas) é Preciso

**DOI:** 10.36660/abc.20220255

**Published:** 2022-05-04

**Authors:** Minna Moreira Dias Romano

**Affiliations:** 1 Faculdade de Medicina de Ribeirão Preto USP São Paulo SP Brasil Faculdade de Medicina de Ribeirão Preto – USP, São Paulo, SP – Brasil

**Keywords:** Cardiomiopatia Hipertrófica Obstrutiva/genética, Técnicas de Ablação/métodos, Ablação por Cateter/métodos, Ablação Septal Alcoólica/métodos, Miectomia, Gradiente

A Cardiomiopatia hipertrófica obstrutiva é a doença cardíaca genética mais comum e, além de seu risco arrítmico, é responsável por sintomas como palpitações, síncope, dispneia de esforços e *angina pectoris* . Dez por cento dos pacientes são refratários ao tratamento clínico com betabloqueadores ou bloqueadores de canais de cálcio. Alternativas de tratamento anatômico como a miectomia cirúrgica (MC) ou outros métodos não invasivos que objetivam a redução do gradiente obstrutivo podem ser considerados em alguns casos.^1 , 2^

Embora a MC seja considerada segura e procedimento de escolha em centros de experiência, ela ainda traz risco de complicações pós-operatórias tais como tamponamento cardíaco, comunicações interventriculares, acidentes cerebrovasculares, dissecção de coronárias, e parada cardíaca não fatal. Permanecem algumas controvérsias sobre opções de tratamentos não invasivos e a experiência do *Heart Team* local é um fator importante nas decisões.^3^

A ablação septal alcoólica (ASA), desenvolvida em 1995, tem sido o procedimento não invasivo mais utilizado e é considerado seguro.^4^ A exequibilidade da ASA está relacionada com a anatomia coronária e o procedimento é considerado como sendo, em geral, responsável por áreas maiores de necrose miocárdica e pode se acompanhar de riscos de distúrbios de condução, bloqueios atrioventriculares e maior risco de dependência de marca-passo artificial. Ainda, a redução média de gradiente obstrutivo após a ASA costuma ser menos significativa do que aquela atingida com a MC. Outras opções de redução septal tais como oclusões coronarianas por *coils* também já foram descritas, embora com riscos de outras complicações miocárdicas.^5^ A ablação por radiofrequência (ARF) é outra opção de tratamento não invasiva, tendo sido mais aplicada em populações pediátricas, dado seu maior risco de arritmias com o uso da ASA.

No volume atual dos ABC, Valdigen et al.,^6^ reportam uma série de casos de 12 pacientes adultos tratados com ARF, guiada pela ecocardiografia transesofágica. Essa modalidade de imagem cardíaca tem boa acurácia para visualizar a localização da ponta do cateter, guiando-a até a região mais obstrutiva do septo interventricular. Os resultados foram bons, com redução significativa dos gradientes médios em via de saída de VE a partir do terceiro mês de seguimento. Dois pacientes apresentaram BRE novo, nenhum deles necessitando de outra intervenção. Não foram observadas complicações maiores.^6^

**Figura 1:**
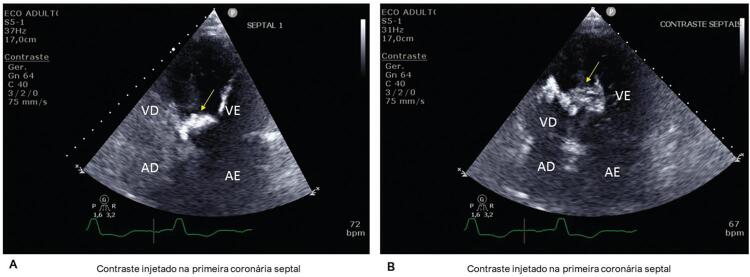
– Imagens usando agente de contraste ecocardiográfico (SonoVue), injetado na primeira septal (Painel A) e na segunda septal (Painel B), setas amarelas. Considerando que a área de miocárdio perfundido pela segunda septal era maior, envolvendo a banda moderadora de VD, a decisão foi feita pela oclusão alcoólica da primeira septal. AD: átrio direito; AE: átrio esquerdo; VD: ventrículo direito; VE: ventrículo esquerdo.

A ecocardiografia intra-procedimento hemodinâmico pode também ser usada para guiar a ASA, tanto com a modalidade transtorácica quanto transesofágica. Recentemente, o uso de agentes de contraste ecocardiográficos, baseados em microbolhas, mostrou-se uma ferramenta alternativa para estudar a perfusão miocárdica relacionada a cada coronária septal. Uma pequena quantidade de contraste é infundida diretamente em cada septal analisada e as imagens de perfusão miocárdica podem ser geradas tanto nas modalidades bidimensional (2D) quanto tridimensional (3D). Com essa ferramenta a decisão acerca da melhor septal a ser abordada pode ser facilitada ( Figura 1 ), ou mesmo a decisão de não proceder com a ASA, muitas vezes porque a irrigação envolve a banda moderadora do VD ou mesmo sua parede livre.^7^ O uso da ecocardiografia 3D parece ser mais acurado do que o da 2D para guiar, de modo seguro, a escolha da coronária alvo^7^ e seu uso deve ser preferido quando disponível.^1^ O uso de contraste ecocardiográfico também pode ser feito na programação da MC.

Em recente metanálise de estudos não randomizados, Bytyci et al.,^8^ concluíram que ASA e MC tem riscos similares de mortalidade, sendo ambos considerados igualmente seguros. Complicações periprocedimento são menores na ASA, mas o risco de necessidade de reintervenção ou de marca-passo é um pouco maior.^8^ Ainda não há dados científicos definitivos comparando opções percutâneas com MC depois da disponibilidade de técnicas avançadas de imagem ecocardiográfica tais como a 3D ou o uso de contrastes de perfusão.

Considerada então a falta de estudos randomizados e controlados neste cenário, embora a MC seja preferida em centros especializados, opções terapêuticas não invasivas podem ser escolhas, a depender de situações individuais de cada caso, considerando riscos e benefícios de cada técnica. O uso de modalidades e técnicas de imagem como a ecocardiografia transesofágica, 3D e com contraste pode ser a peça que faltava para o sucesso de algumas técnicas.
