# Supplementary material for: Ecocardiografia Guiando Tratamento Percutâneo de Cardiomiopatia Hipertrófica Obstrutiva: Navegar (em Águas Conhecidas) é Preciso
Source: Arq Bras Cardiol. 2022 May 4;118(5):873–4. [Article in Portuguese] doi: 10.36660/abc.20220255 (PMC9368878; doi:10.36660/abc.20220255)
Supplement: Translation

# Translation: Echocardiography Guiding Percutaneous Treatment of Obstructive Hypertrophic Cardiomyopathy: Navigating (In Known Waters) Is Necessary

**Authors:** Minna Moreira Dias Romano

**Affiliations:** 1 Brazil Faculdade de Medicina de Ribeirão Preto – USP, São Paulo, SP – Brazil

**Keywords:** Cardiomyopathy, Hypertrophic/genetics, Ablation Techniques/methods, Alcohol Septal Ablation/methods, Miectomy, Gradient

Obstructive hypertrophic cardiomyopathy (HOCM) is the most common cardiac genetic disease and, besides the risk of malignant arrhythmias, it is responsible for symptoms such as palpitations, syncope, or presyncope, exertional dyspnea, and angina pectoris. Ten percent of patients are refractory to clinical treatment with beta-blockers or calcium channel blockers. Treatment alternatives such as surgical myectomy (SM) or non-invasive methods to reduce left ventricle outflow obstruction may be considered in this population.^1 , 2^

Although surgical myectomy is considered safe and the procedure of choice in experienced centers, it still has a percentage of post-procedure complications such as tamponade, interventricular septal communication, cerebrovascular accident dissection of coronary arteries, and non-fatal cardiac arrest. Controversy still exists about non-surgical hemodynamic treatment options, and the Heart Team’s expertise is an important factor regarding decisions.^3^

Alcohol septal ablation (ASA), developed in 1995, has been the most frequently used non-surgical procedure, considered safe.^4^ ASA feasibility is related to coronary septal anatomy and is reported to be responsible for relatively big areas of myocardium damage and carries a risk of bundle branch block, atrioventricular block, and pacemaker dependency. Also, gradient reductions after ASA used to be less impressive than surgical ones. Other options of septal occlusion as coiling septal arteries have also been described, although with other related complications.^5^ Radiofrequency ablation (RFA) is another option for treating HOCM and was mostly applied to children based on this population’s higher risk of arrhythmias when submitted to alcoholization.

In this volume of ABC, Valdigen et al.^6^ reported a case series of 12 adult patients treated with RFA guided with transesophageal echocardiography. This imaging modality can accurately visualize the catheter location in LV and help position it after evaluating the most obstructive portion of the LV septum. Results were good, with a significant reduction of LVOT obstruction from 3 months of follow-up. Two patients developed LVBBB without any interventional need. No major complications occurred.^6^

Intraprocedural echocardiography can also guide septal alcoholization using transthoracic or transesophageal views. Recently, echocardiographic enhanced contrast agents, based on microbubbles, has been used as a tool to study and quantify myocardium perfused areas related to septal coronaries. A small amount of contrast agents is infused on isolated septal coronaries, and perfusion myocardium images can be generated from bidimensional or even tridimensional techniques. With this tool, deciding the best coronary to approach ( Figure 1 ) or even to decline proceeding is easier.^7^ The use of three-dimensional echocardiography seems to be more accurate than conventional bidimensional to target safe and long-term effective septal reduction with ASA in obstructive HCM,^7^ and its use should be preferred when available.^1^ Echocardiographic contrasting of the myocardium can also be evaluated to guide surgical myectomy (SM).

**Figure 1:** – Images using echocardiography enhanced agent (SonoVue) injected at first septal coronary (Panel A) and second septal coronary (Panel B). Once the perfused myocardial area from the second septal coronary was larger and involved the RV’s moderator band, the decision to occlude the first septal coronary was taken. RA: right atria; LA: left atria; RV: right ventricle; LV: left ventricle.

In a recent meta-analysis of non-randomized studies, Bytyci, et al.^8^ conclude that ASA and SM treatment of HOCM carry a similar mortality risk. Peri-procedural complications are less in alcohol ablation, but re-intervention and pacemaker implantations are more common.^8^ There is still no significant data comparing non-surgical options to SM after using advanced echocardiographic techniques with or without perfusion agents.

Once no randomized trial data compares the efficacy of techniques in this scenario, although SM is preferred in experienced centers, treatment choices will still be chosen based on center expertise and heart team considerations about individual risk and benefits. The use of imaging techniques such as echocardiography, which can help guide the best anatomical septum target, maybe is the missing piece for success.
